# COVID-19 Vaccination Intent and Belief that Vaccination Will End the Pandemic

**DOI:** 10.3201/eid2808.212556

**Published:** 2022-08

**Authors:** Marion de Vries, Liesbeth Claassen, Mattijs Lambooij, Ka Yin Leung, Kees Boersma, Aura Timen

**Affiliations:** National Institute for Public Health and the Environment, Bilthoven, the Netherlands (M. de Vries, L. Claassen, M. Lambooij, K.Y. Leung, A. Timen);; Vrije Universiteit, Amsterdam, the Netherlands (K. Boersma, A. Timen)

**Keywords:** COVID-19, coronavirus disease, vaccination, vaccination intent, perception, beliefs, behavior, understanding, respiratory infections, pandemic, zoonoses, the Netherlands

## Abstract

High vaccination coverage is considered to be key in dealing with the coronavirus disease (COVID-19) pandemic. However, vaccine hesitancy can limit uptake. We examined the specific coronavirus beliefs that persons have regarding COVID-19 and COVID-19 vaccines and to what extent these beliefs explain COVID-19 vaccination intentions. We conducted a survey among 4,033 residents of the Netherlands that examined COVID-19 vaccination intentions and various beliefs. Random forest regression analysis explained 76% of the variance in vaccination intentions. The strongest determinant in the model was the belief the COVID-19 crisis will only end if many persons get vaccinated. Other strong determinants were beliefs about safety of vaccines, specifically in relation to vaccine development and approval process; (social) benefits of vaccination; social norms regarding vaccination behavior; and effectiveness of vaccines. We propose to address these specific beliefs in communications about COVID-19 vaccinations to stimulate vaccine uptake.

The COVID-19 pandemic has profoundly affected global health and well-being. Since 2020, countries worldwide have experienced high rates of illness and death caused by COVID-19, and many societies have dealt with often stringent outbreak control measures. The successful development of effective vaccines has provided a much-wanted major step toward controlling the pandemic. However, for the vaccines to be successful during outbreak control, a high and equally distributed vaccine uptake is essential. Next to possible barriers of limited COVID-19 vaccine availability and accessibility, vaccine hesitancy can also form a considerable barrier to reaching a high vaccine uptake.

The public acceptance of vaccines has been a global concern for decades. Before the COVID-19 crisis, in 2019, the World Health Organization declared vaccine hesitancy as one of the top 10 global public health threats (*1*). Vaccine hesitancy has been defined as a broad range of vaccine-related attitudes and behavior, from having some doubts and delaying vaccinations up to complete refusal of vaccines (*2*). Various studies have provided insights into beliefs underlying vaccination hesitancy and vaccination intentions for childhood vaccinations (*3**–**5*); influenza vaccinations (*5*,*6*), including pandemic influenza A(H1N1) vaccination (*5**–**7*); and COVID-19 vaccinations (*8**–**12*). Personal beliefs that are known to have a major role in vaccination decision-making are beliefs about the need for, safety of, and effectiveness of vaccines.

Many studies that examine determinants of vaccination hesitancy, intentions, or behavior (e.g., studies applying the health belief model [*9**–**13*]) explore beliefs in relatively general terms. For example, surveys may simply ask respondents whether they have concerns about the safety of vaccines. It is useful to have more detailed knowledge of these beliefs for 3 reasons. First, in-depth insights into beliefs can provide more concrete input toward developing well-adapted communication (*14*). For example, concerns about safety of vaccines might be related to beliefs about the vaccine production process, long-term side-effects, and composition of vaccines. Such specific beliefs should be addressed in communications. Second, COVID-19 vaccination intentions are likely to be associated with specific beliefs that differ from those found in research during other vaccination campaigns. This reaction might be the case for beliefs about the rapid vaccine development process, the new technologies used (mRNA), and the personal freedom associated with vaccination (through vaccination entry passes). Third, there might be major differences in (the influence of certain) beliefs underlying vaccination decisions between countries or communities (*15*) (e.g., due to differences in experiences with the COVID-19 pandemic, information streams, and vaccination campaign history).

Consistent with earlier research on vaccination decisions (*16*), we adopted a mental models perspective in studying beliefs underlying COVID-19 vaccination intentions among persons in the Netherlands (*14*). This perspective entails a detailed study of interrelated beliefs of a subject, in this case COVID-19 and the COVID-19 vaccinations. These beliefs form a mental model underlying decisions of persons regarding COVID-19 vaccination. By gaining in-depth insights into these various beliefs, we can identify knowledge gaps and misbeliefs that need to be addressed in communications. In addition, by studying which beliefs are useful determinants of vaccination intentions, we aimed to identify beliefs that should be addressed and prioritized in communications to optimize vaccine acceptability and uptake.

## Methods

### Study Population and Procedure

We conducted an online survey during March 12–22, 2021. At that time, 1.5 million of the 17.5 million residents of the Netherlands were partly or fully vaccinated against COVID-19 (data from March 14, 2021) (*17*). We sent the survey (in Dutch) to 6,810 persons in the Netherlands (>18 years of age) by using an online survey panel (ISO/IEC 27001:2013; I&O Research, https://www.ioresearch.nl). Members from this survey panel were recruited by using random samples of name and address data. The sample invited for participation to this survey was selected to be representative of the general population of the Netherlands (>18 years of age) on the basis of demographic characteristics.

Panel members provided informed consent for participation to the survey panel. Survey completion took ≈10‒15 minutes. The Clinical Expertise Centre at the National Institute for Public Health and the Environment reviewed the study protocol and determined that the study was exempt from needing further approval from an ethics research committee (study no. LCI-485).

### Development of Survey Measurements

We measured vaccination intention as follows. All respondents who indicated to have received an invitation for a COVID-19 vaccination but who had not yet been vaccinated were asked, “Do you want to get vaccinated against the corona virus?” Respondents who indicated not yet to be invited for a COVID-19 vaccination were asked, “If you are invited for a COVID-19 vaccination, do you then want to get vaccinated?” Both questions could be answered on a 5-point Likert scale: 1. Certainly not; 2. Probably not; 3. Don’t know; 4 Probably yes; 5. Certainly yes. The answers to both questions were taken together in 1 variable that was called COVID-19 vaccination intention.

To identify the beliefs about COVID-19 and the COVID-19 vaccines that should be assessed in this study, we studied literature on sociopsychological determinants of vaccination intentions to identify main elements in mental models of persons underlying COVID-19 vaccination intentions (*2*,*4*,*18*,*19*). In addition, we used recent qualitative studies (survey open answer categories and in-depth interviews) on COVID-19 vaccination acceptability among members of the public in the Netherlands (*20*,*21*) to identify specific beliefs within the familiar mental model themes and to identify beliefs that might not have been identified in studies on other vaccination campaigns.

We studied beliefs about COVID-19 and COVID-19 vaccines by using this question: “We would like to know what you think about the corona virus/vaccination against the corona virus. For each statement, indicate to what extent it aligns with what you think. I think ….” The question was followed by 25 statements that were scored on a 5-point Likert scale (1 = certainly not to 5 = certainly yes) (Table 1). The 25 beliefs can be categorized into 7 elements of mental models of persons, namely beliefs about risk for COVID-19 regarding oneself, risk for COVID-19 regarding one’s loved ones, safety of vaccination, effectiveness of vaccination, (social) benefits of vaccination, alternatives to vaccination, social norms regarding vaccination behavior, and accessibility of vaccination.

**Table 1:** Survey questions and items addressing beliefs about COVID-19 and the COVID-19 vaccines among persons in the Netherlands*

Elements of mental models	Survey items (1–25): I think…	Summarized item	% Responses/answer category,† n = 3,628	Mean (+SD) response score	Pearson correlation for vaccination intention‡
Risk perceptions COVID-19: self	1. …the chance is high that I will be infected with coronavirus.	COVID, high likelihood infection: self	1. 10.1	2.9 (1.0)	0.1
			2. 26.9		
			3. 38.3		
			4. 17.8		
			5. 6.9		
	2. …I could get seriously ill if I get infected with coronavirus.	COVID, possibility of severe illness: self	1. 14.3	3.0 (1.3)	0.3
			2. 24.0		
			3. 26.6		
			4. 21.0		
			5. 14.1		
Risk perceptions COVID-19 loved ones	3. …the chance is high that someone from my family or friends will get infected with coronavirus.	COVID, high likelihood of infection: loved ones	1. 4.0	3.5 (1.1)	0.1
			2. 12.4		
			3. 36.2		
			4. 27.1		
			5. 20.4		
	4. …someone from my family or friends could become seriously ill from being infected with coronavirus.	COVID, possibility of severe illness: loved ones	1. 2.8	3.9 (1.1)	0.3
			2. 8.7		
			3. 23.8		
			4. 30.0		
			5. 34.6		
	5. …the chance is high that I will infect others if I become infected with coronavirus myself.	COVID, likelihood of infecting others	1. 8.2	3.3 (1.2)	0.2
			2. 20.4		
			3. 25.2		
			4. 25.6		
			5. 20.6		
Safety vaccination	6. …the chance is high to experience mild side effects due to vaccination against coronavirus.	Vaccination, high likelihood of mild side effects	1. 4.3	3.6 (1.1)	−0.1
			2. 12.9		
			3. 27.3		
			4. 33.1		
			5. 22.4		
	7. …I could get seriously ill from the coronavirus vaccine.	Vaccination, possibility of severe illness	1. 34.7	2.2 (1.2)	−0.6
			2. 31.2		
			3. 20.1		
			4. 8.8		
			5. 5.2		
	8. …side effects of the coronavirus vaccination are well researched.	Vaccination, side effects well researched	1. 13.0	3.3 (1.3)	0.7
			2. 14.7		
			3. 19.7		
			4. 32.4		
			5. 20.3		
	9. …the vaccines against the coronavirus have been developed too quickly.	Vaccination, developed too quickly	1. 22.5	2.7 (1.3)	−0.6
			2. 25.7		
			3. 23.4		
			4. 13.4		
			5. 14.9		
	10. …the new techniques used to make coronavirus vaccines are safe.	Vaccination, new techniques are safe	1. 7.0	3.6 (1.1)	0.7
			2. 8.6		
			3. 26.7		
			4. 34.5		
			5. 23.2		
	11. …the vaccine against coronavirus can reduce one's fertility.	Vaccination, reduces fertility	1. 37.3	2.2 (1.1)	−0.5
			2. 17.9		
			3. 36.7		
			4. 5.1		
			5. 3.0		
	12. …if a vaccination is approved for the Dutch market, it is safe.	Vaccination, approved therefore safe	1. 7.2	3.7 (1.2)	0.7
			2. 10.9		
			3. 15.4		
			4. 35.7		
			5. 30.8		
Effectiveness vaccination	13. …the coronavirus vaccine protects me well against coronavirus disease.	Vaccination, good protection	1. 5.2	3.8 (1.1)	0.7
			2. 7.9		
			3. 17.3		
			4. 43.8		
			5. 25.7		
	14. …the vaccination only protects against illness from coronavirus for a short time.	Vaccination, only short protection	1. 6.3	3.1 (1.0)	−0.3
			2. 17.4		
			3. 47.6		
			4. 21.6		
			5. 7.2		
	15. …you can infect others with coronavirus if you are vaccinated.	Vaccination, possible of infecting others	1. 10.9	3.0 (1.1)	−0.3
			2. 20.7		
			3. 38.8		
			4. 19.3		
			5. 10.4		
Social benefits vaccination	16. …I can live sooner without coronavirus measures if I get vaccinated against coronavirus.	Vaccination, live longer without measures	1. 8.8	3.8 (1.3)	0.6
			2. 6.6		
			3. 15.8		
			4. 30.0		
			5. 38.8		
	17. …I can go abroad sooner if I get vaccinated against coronavirus.	Vaccination, can go abroad sooner	1. 7.7	3.8 (1.2)	0.3
			2. 6.5		
			3. 17.6		
			4. 30.6		
			5. 37.6		
	18. …the crisis will only end if many persons vaccinated.	Vaccination, end of crisis	1. 7.6	4.1 (1.2)	0.7
			2. 5.5		
			3. 9.9		
			4. 27.8		
			5. 49.3		
Alternatives to vaccination	19. …I'm already immune to coronavirus disease.	COVID, already immune	1. 48.1	2.0 (1.1)	−0.3
			2. 20.3		
			3. 21.7		
			4. 6.8		
			5. 3.1		
	20. …my good health protects me from coronavirus.	COVID, protected by good health	1. 20.3	2.8 (1.2)	−0.3
			2. 20.6		
			3. 29.7		
			4. 21.9		
			5. 7.5		
	21. …there are already enough other drugs that cure disease caused by coronavirus.	Vaccination, other drugs are available	1. 47.6	1.9 (1.0)	−0.5
			2. 27.1		
			3. 18.2		
			4. 4.1		
			5. 2.9		
Social norms for vaccination behavior	22. …most of my friends and family will be vaccinated against coronavirus.	Vaccination. behavior of loved ones	1. 2.7	4.2 (1.0)	0.6
			2. 3.8		
			3. 13.2		
			4. 35.2		
			5. 45.1		
	23. …most of my friends and family expect me to be vaccinated against coronavirus.	Vaccination, expectations of loved ones	1. 6.1	4.0 (1.2)	0.7
			2. 4.9		
			3. 17.3		
			4. 27.5		
			5. 44.2		
	24. …most persons in the Netherlands will be vaccinated against coronavirus.	Vaccination, behavior of public in the Netherlands	1. 1.2	4.0 (0.8)	0.3
			2. 2.5		
			3. 16.3		
			4. 51.7		
			5. 28.3		
Accessibility vaccination	25. …it will take me a lot of time or effort to get vaccinated against coronavirus.	Vaccination, takes time and effort	1. 48.5	2.0 (1.2)	−0.3
			2. 23.6		
			3. 15.6		
			4. 7.1		
			5. 5.2		

*Corresponds to subsequent Tables and Figures. †Responses used a 5-point Likert scale: 1, certainly not; 5. certainly yes. ‡p<0.001 by Pearson correlation (2-tailed) test with vaccination intention for all elements listed.

### Analyses

We analyzed on responses of (at that time) unvaccinated respondents. Descriptive analyses were performed for vaccination intention and the 25 beliefs about COVID-19 and the vaccines. In addition, we calculated Pearson correlations (2-tailed) between vaccination intention and all 25 beliefs and between all beliefs separately.

We subsequently performed a regression analysis by using Random forest (RF) (*22*) in R (*23*) to assess the extent to which beliefs explain variance in vaccination intentions, and to identify which specific beliefs are good determinants of vaccination intentions (Appendix). RF is a machine learning method for regression and classification based on an ensemble of decision trees. This method makes no assumptions about data distribution and is well suited to address 3 complicating features of the study responses for analyses: a dependent variable (COVID-19 vaccination intention) that is not normally distributed, many (partly intercorrelated) independent variables (beliefs), and potentially nonlinear relationships between independent variables and the dependent variable. The RF method has been successfully applied in explaining vaccination intentions (*16*) and screening intentions (*24*). We controlled the RF regression analysis for age, sex, education level, region of residence, migration background, health status, previous coronavirus infection, being invited for a COVID-19 vaccination, perceived allergy for vaccinations, employment in healthcare, and religious motivations (Appendix) by adding these as independent variables to the RF model.

We considered 4 types of output from the RF regression analyses. The first output was the variable importance ranking (VIR), which ranks independent and control variables in terms of how much these contribute to explaining the dependent variable. The second output was the partial dependence (also known as marginal means) that indicates the direction and strength of the relationship between the independent and dependent variable. The third output was the cumulative variance explained, which is the variance explained after adding an independent variable to the model in the sequence of the VIR. The fourth output was the total variance explained.

## Results

### Study Population

The survey response rate was 59% (n = 4.033). The survey population was reasonably comparable to the general population in the Netherlands (>18 years of age) for demographic characteristics (Table 2).

**Table 2:** Variables for study population testing major role for the belief that COVID-19 vaccination will end the pandemic, the Netherlands*

Variable	Answer categories	No. (%)
Sex	M	1,991 (49)
	F	2,042 (51)
Age, y	18–29	560 (14)
	30–39	503 (12)
	40–49	574 (14)
	50–59	765 (19)
	60–69	805 (20)
	70–79	613 (15)
	>80	213 (5)
Level of education	Low	824 (20)
	Moderate	1,535 (38)
	High	1,674 (42)
Region of residence in the Netherlands	West	1,736 (43)
	North	470 (12)
	East	866 (22)
	South	961 (24)
Migration background	None	3,267 (81)
	Western	444 (11)
	Other	306 (8)
	Unknown	16 (0)
Invited for a COVID-19 vaccination	Invited	642 (16)
	Not (yet) invited	3,391 (84)
Vaccinated against COVID-19	Vaccinated	405 (10)
	Not (yet) vaccinated	3,628 (90)
Total	NA	4,033 (100)

*COVID-19, coronavirus disease; NA, not applicable.

### COVID-19 Vaccination Intention

Most (2,266/3,628, 62.5%) unvaccinated respondents indicated that they would certainly want to get vaccinated against COVID-19, 645 (17.8%) would probably want to get vaccinated, 257 (7.1%) did not know (yet), 213 (5.9%) would probably not want to get vaccinated, and 247 (6.8%) indicated certainly not. The mean (+SD) response of vaccination intention was 4.2 (+1.2).

### Beliefs about COVID-19 and COVID-19 Vaccines

**Figure 1:**
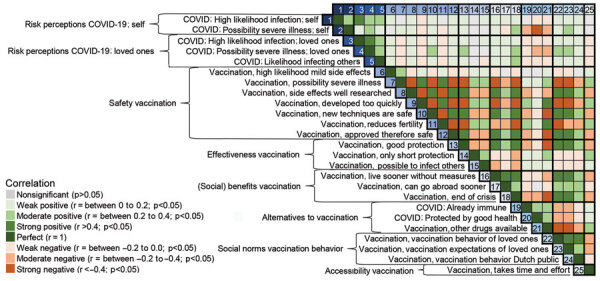
COVID-19 vaccination intent and belief that COVID-19 vaccination will end the pandemic among persons in the Netherlands. Pearson correlation matrix (2-tailed) heat map with all beliefs about COVID-19 and COVID-19 vaccinations was visualized per mental models element (risk perceptions COVID-19: self, risk perceptions COVID-19: loved ones, safety vaccination, effectiveness vaccination, (social) benefits vaccination, alternatives to vaccination, social norms vaccination behavior, accessibility vaccination). For a more detailed correlation matrix, see Appendix.

Descriptive statistics and Pearson correlations (2-tailed) with vaccination intention showed that all 25 beliefs were significantly (p<0.001) correlated with vaccination intention (Table 1). Correlations between different beliefs about COVID-19 and COVID-19 vaccinations (Figure 1) showed moderate-to-strong correlations between different risk perception beliefs of COVID-19 (for self and loved ones), and, at the same time, mostly weak correlations between these COVID-19 beliefs and the several beliefs about COVID-19 vaccinations. In addition, we observed many strong correlations between the various beliefs about COVID-19 vaccinations, especially in relation to beliefs about the safety of vaccination.

Personal risk perceptions of respondents for COVID-19 were moderate, with values of 2.9 (+1.0) for the belief about high likelihood of infection and 3.0 (+1.3) for the belief about possibility of severe illness. For loved ones, these COVID-19 risk perceptions were scored relatively higher: 3.5 (+1.1) for high likelihood of infection and 3.9 (+1.1) for possibility of severe illness. Respondents valued the likelihood to infect others if infected themselves with a value of 3.3 (+1.2).

Safety of vaccinations was generally trusted by respondents (Table 3), but some notable variations in responses were observed. For example, 27.7% of respondents indicated not believing that the side effects of vaccinated were well researched (mean 3.3, SD +1.3), and 28.3% of respondents believed that the vaccines were developed too quickly (mean 2.7, SD +1.3). With regard to the effectiveness of vaccinations, respondents believed that vaccines would protect them well against COVID-19 (mean 3.8, SD +1.1). Respondents seemed somewhat unsure about whether vaccines only protect for a short while (mean 3.1, SD +1.0) and whether one can still infect others after vaccination (mean 3.0, SD +1.1). At the time of data collection, scientific knowledge about those last 2 vaccine aspects was also limited.

**Table 3:** Cumulative variance explained and partial dependence of 10 strongest determinants of COVID-19 vaccination intention in random forest model for residents of the Netherlands

Ten strongest determinants in random forest model	Cumulative variance explained, %	Partial dependence, lowest–highest value*	Direction of relationship with vaccination intention
Vaccination, end of crisis	54	3.9–4.3	Positive
Vaccination, expectations of loved ones	62	4.0–4.4	Positive
Vaccination, developed too quickly	70	4.5–4.2	Negative
Vaccination, side effects well researched	72	4.1–4.3	Positive
Vaccination, approved therefore safe	72	3.9–4.2	Positive
Vaccination, good protection	73	4.2–4.5	Positive
Vaccination, new techniques are safe	73	4.2–4.4	Positive
Vaccination, live sooner without measures	74	4.2–4.3	Positive
Vaccination, behavior of loved ones	74	4.0– 4.3	Positive
Vaccination, possibility severe illness	74	4.4–4.3	Negative

*Interpretation of the partial dependence figures, first row (end of crisis) as an example: When all other determinants are kept constant, the lowest value for this belief (1, certainly not) matches a mean vaccination intention of 3.9 and the highest value of this belief (5, certainly yes) matches a mean vaccination intention of 4.3. Because many of the beliefs correlate strongly, and the partial dependence figures are controlled for all other determinants in the model, the partial dependence ranges are small. Partial dependence figures corresponding to all of the 10 best determinants values (1–5) are shown in the Appendix.

In terms of (social) benefits of vaccinations, vaccinations were commonly seen as the only way out of the COVID-19 crisis (mean 4.1, SD +1.2) and as a means to return to a life without COVID-19 restrictions sooner (mean 3.8, SD +1.3). With regard to alternatives to vaccination, respondents generally did not believe that there were (sufficient) drugs that could cure COVID-19 (mean 1.9, SD +1.0), and few respondents believed that they were immune to COVID-19 (mean 2.0, SD +1.1). Some support was found for the belief that one’s good health would protect against COVID-19 (mean 2.8, SD +1.2).

We found that perceived social norms were generally in favor of vaccination. Beliefs that friends and family expected that the respondent would get vaccinated (mean 4.0, SD +1.2), and the beliefs that most of the respondent’s family and friends (mean 4.2, SD +1.0), as well as most residents in the Netherlands (mean 4.0, SD +0.8), would get vaccinated were largely supported. Accessibility of vaccination did not seem a large obstacle because the belief that getting vaccinated would take a lot of time or effort was not strongly supported among the respondents (mean 2.0, SD +1.2).

### Variance in COVID-19 Vaccination Intention

**Figure 2:**
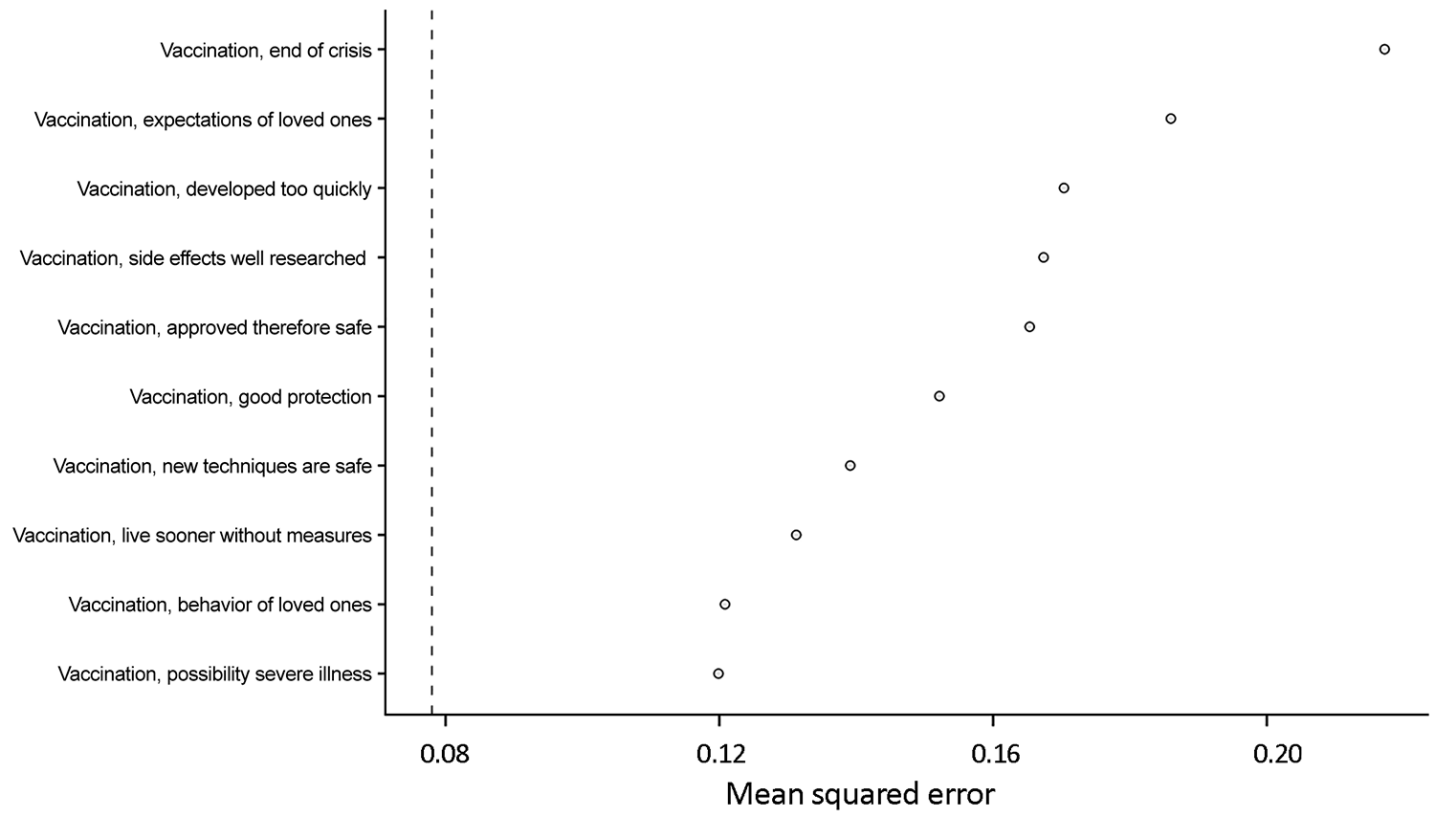
COVID-19 vaccination intent and belief that COVID-19 vaccination will end the pandemic among persons in the Netherlands. Variable ranking random forest model shows the 10 strongest determinants. n = 3,614, explained variance 0.76, mean squared error 0.078 (dashed vertical line).

The random forest model explained 76% of the variance in COVID-19 vaccination intentions. This analysis was performed using data for 3,614 of the 3,628 unvaccinated respondents (14 respondents were excluded from the analysis because of missing values). We provide the VIR with the 10 best explaining beliefs (Figure 2). We also provide the cumulative variances explained and partial dependence of the 10 best explaining beliefs (Table 3). Of these 10 best explaining beliefs, 5 beliefs concern safety of vaccinations, 2 beliefs are about social benefits of vaccination, 2 beliefs concern social norms regarding vaccination behavior, and 1 belief is about effectiveness of vaccination.

There was no clear selection of the 25 beliefs that explained variance in vaccination intention considerably more strongly than all other beliefs. Instead we observe a gradual progression in explanatory value of various beliefs (VIR with all determinants; Appendix). Because there are many intercorrelations between the beliefs (Figure 1), and many of the beliefs are associated with vaccination intentions, the partial dependence ranges were also small (Table 3). Our findings confirm that vaccination decisions are made on the basis of a complex web of interrelated beliefs (mental models), rather than on a few independent perceptions. Although a small number of these beliefs can (statistically) explain a large part of the variance in vaccination intentions, one needs to keep in mind that in reality beliefs never stand on their own. This said, the belief “the corona crisis will only end if many people get vaccinated” seems, distinctively, the strongest determinant in the model. By adding only this variable to the model, we can explain 54% of the variance in vaccination intentions.

We conducted sensitivity analyses in which we repeated the main RF analyses for 3 age groups (18–34 years, 35–64 years, and >65 years). We repeated the main analysis with a binary dependent variable, explaining differences between those with low vaccination intentions (original values 1 and 2) and those who were unsure (value 3). Results were consistent with those of the main analyses and did not affect our conclusions.

## Discussion

Our findings provide detailed insights into COVID-19 vaccination intentions and the underlying beliefs about COVID-19 and the COVID-19 vaccines among residents in the Netherlands during 2021. No major knowledge gaps or misbeliefs were observed, but we did observe some considerable concerns with regard to the vaccine development and approval process. The beliefs assessed in our study explained a large part of the variance in COVID-19 vaccination intentions. Beliefs about the safety of vaccines, (social) benefits of vaccination, social norms regarding vaccination behavior and the effectiveness of vaccines were, relative to other beliefs, strong determinants of vaccination intention for persons. The strongest determinant in the model was the belief “the corona crisis will only end if many people get vaccinated.”

Our study results showed strong beliefs, and the explanatory value of these beliefs, about (social) benefits after being vaccinated or reaching a high vaccination coverage. The belief that the COVID-19 crisis will only end if many persons get vaccinated could (statistically) explain more than half of the variance in COVID-19 vaccination intentions. It is striking that this belief seemed to be, at least somewhat, a better determinant of vaccination intentions than beliefs about personal protection against the vaccine-preventable disease or beliefs about safety of vaccines, which have often been identified as the most essential psychosocial determinants of vaccination intentions (*5*,*17*,*18*). The wish for relaxation of COVID-19 control measures and for the ending of the enduring crisis seem to have been stronger among many than the wish for personal protection against disease (although these wishes are not mutually exclusive). This finding might be explained by the considerable effect of COVID-19 measures on lives of persons (*25*) and the observed moderate COVID-19 risk perceptions. Our results also suggest that persons who did not believe that high vaccination coverage is the only solution to end the COVID-19 crisis were not less likely to vaccinate.

We might expect that over time fewer persons will have the belief of vaccination being the only solution to end the crisis, during the winter of 2021, when lockdown measures were again necessary, despite relatively high vaccination coverage (*26*). A decrease in this belief might lead to a decrease in vaccination acceptability. In communications, we are faced with a dilemma. In the short run, providing clear future perspectives regarding personal and societal benefits after reaching a high vaccination coverage, might considerably help in motivating persons to get vaccinated. At the same time, transparency about uncertainties regarding these perspectives are necessary from an ethical point of view, but also to prevent disappointments in the future resulting from too optimistic expectations. Transparency is also crucial in remaining trust and support for control measures (*27*,*28*).

Consistent with previous research, we found that various beliefs about the safety of the vaccines were major determinants of COVID-19 vaccination intentions (*12*,*29*). Five of the 10 major explanatory beliefs were related to safety. Four of these 5 beliefs were about vaccine development and approval processes. Rapid development of vaccines and the approval process, and the use of new techniques (e.g., mRNA vaccines) have probably increased public concerns about vaccine safety. Concerns about rapid development of vaccines were also observed in previous research on COVID-19 vaccination perceptions (*8*) and pandemic influenza A(H1N1) virus vaccination perceptions (*30*). Authorities must provide persons with timely and transparent information about development, approval, and safety monitoring of COVID-19 vaccines to fulfil their information needs. If such information is not, or is scarcely, provided by authorities, persons are likely to search for this information elsewhere on the internet, with the considerable risk that this would lead them to vaccine skeptical sources (*31*,*32*).

We showed that beliefs about descriptive and subjective social norms, specifically with regard to vaccination expectations and behavior of friends and family, were also major determinants of COVID-19 vaccination intentions. The role of social norms in vaccination behavior was also suggested in previous research with regard to COVID-19 vaccinations (*8*,*33*), influenza vaccinations (*34*,*35*), and human papillomavirus vaccinations (*36*,*37*). These findings suggest the potential for interventions focused on endorsing social norms with regard to vaccination (e.g., providing narratives in communication materials for peers who vaccinated). In addition, this finding might indicate that persons are, at least partly, segregated in like-minded groups on the basis of COVID-19 vaccination intentions, which could increase the risk for local outbreaks.

Beliefs about the health risks posed by COVID-19 were not found among the major determinants of vaccination intentions. A similar result was seen in a study on meningococcal vaccination intentions that had a similar study approach (*16*). An explanation for this result can lie in the distribution of responses in vaccination intentions. Because most of our respondents intended to vaccinate against COVID-19, the explanatory analysis shows mainly how persons who are not (so) willing to vaccinate differ from those who do want to vaccinate, because that is where the variance in responses can be found. Perceptions of the health risk posed by COVID-19 are likely major reasons for persons to vaccinate but might not be among the most essential reasons for those who do not intend to vaccinate.

The first limitation of our study is that, although the study population is at large fairly comparable with the population in the Netherlands in terms of main demographic characteristics, it is not a perfect representation. Presumably, there is an overlap between those persons who are difficult to reach for vaccination with those persons who are difficult to reach for research purposes. Second, our study was cross-sectional and conducted in a period that had rapid developments in information about COVID-19 vaccinations. For example, just before the start of our data collection, Denmark announced suspending use of vaccine from AstraZeneca (https://www.astrazeneca.com) after reports of possible severe adverse events; during the second half of our data collection, the Netherlands also temporarily suspended these vaccinations (*38*). Such developments might have affected the outcomes of our study (e.g., through a potential decrease in people’s trust in vaccine safety). Also, subsequent events might lead to slightly different results if the study was repeated. It would be highly valuable to repeat research like ours throughout prolonged crises and in multiple settings to monitor changes and differences. Third, our study focused on beliefs about COVID-19 and COVID-19 vaccinations to provide concrete input for communication. Our study did not address other possible major determinants of vaccination intentions, such as trust in institutions or health literacy. Fourth, we did not include beliefs about conspiracy theories in this study, which in hindsight could have added interesting insights. Such beliefs were not included because these were not pronounced in the literature nor in the qualitative data at the time we developed our survey.

Results of this study provide several essential key points for future research, policy, and communication. First, COVID-19 vaccinations decisions are not made purely by considering the pros and cons for one’s own health. Other (social) benefits of COVID-19 vaccination, related to the relaxation of COVID-19 control measures, are likely to play a major role in vaccination decisions of persons. Providing clear perspectives with regard to these benefits might increase vaccination uptake. At the same time, it is highly essential to address the uncertainties with regard to those social benefits and to prevent future disappointments and decreases in trust and support. Second, social norms regarding peers have been shown to be an essential factor in COVID-19 vaccination intentions, which suggests the potential for norms to induce interventions to increase vaccination uptake. Future research should focus on the characterization and identification of like-minded social networks who are hesitant to vaccinate against COVID-19 to provide well-tailored interventions. Finally, it is highly essential to provide transparent and accessible information about vaccine development and approval process and the probability of potential adverse events caused by vaccination to address concerns about safety of COVID-19 vaccines.

Appendix Additional information on COVID-19 vaccination intent and belief that COVID-19 vaccination will end the pandemic among persons in the Netherlands.
