# Supplementary material for: COVID-19 Vaccination Intent and Belief that Vaccination Will End the Pandemic
Source: Emerg Infect Dis. 2022 Aug;28(8):1642–9. doi: 10.3201/eid2808.212556 (PMC9328899; doi:10.3201/eid2808.212556)
Supplement: Appendix — Additional information on COVID-19 vaccination intent and belief that COVID-19 vaccination will end the pandemic among persons in the Netherlands. [file 21-2556-Techapp-s1.pdf]

Article DOI: https://doi.org/10.3201/eid2808.212556

COVID-19 Vaccination Intent and Belief that Vaccination Will End the Pandemic

Appendix

Random Forest Analysis Procedure

Random forest (RF) has several hyperparameters that can be chosen by the user. To find the optimal set of hyperparameter values to obtain the RF model to fit our data, we performed a grid search. The hyperparameters that we tuned were the number of features to consider at each node (mtry), the minimum number of nodes per tree (min_n) and the number of trees (trees). Other hyperparameters were set to default as implemented in ranger (*1*) (R version 0.13.1) including sampling with replacement (including bootstrapping). Furthermore, to make optimal use of all data, rather than setting aside a separate test set, we used 10-fold cross-validation (CV). CV is a resampling procedure so that different parts of the data are used as train and test set in each iteration. CV enables to balance the bias-variance trade-off and prevent overfitting the model. The best model, i.e., best set of hyperparameters is selected based on the highest total variance explained, averaged over the 10 different test sets that arise from the CV procedure. By comparing the total variance explained metric across train and test sets in the CV procedure we ensured that the model did not overfit to the data. Finally, the sample size was sufficiently large to obtain a good fit to the data in terms of total variance explained.

Reference

1. Wright MN, Ziegler A. Ranger: a fast implementation of random forests for high dimensional data in C++ and R. J Stat Softw. 2017;77:1–17. https://doi.org/10.18637/jss.v077.i01

Appendix Table 1. Control variables tested by random forest analysis

Variable	Answer categories	Frequency	Percentage
Sex	Male	1,991	49
	Female	2,042	51
Age, y	18–29	560	14
	30–39	503	12
	40–49	574	14
	50–59	765	19
	60–69	805	20
	70–79	613	15
	≥80	213	5
Level of education	Low	824	20
	Moderate	1,535	38
	High	1,674	42
Migration background	None	3,267	81
	Western	444	11
	Other	306	8
	Unknown	16	0
Region of residence in the Netherlands	West	1,736	43
	North	470	12
	East	866	22
	South	961	24
Invited for COVID-19 vaccination	Invited	642	16
	Not (yet) invited	3,391	84
Employment in healthcare	No	3,553	88
	Yes, as caretaker	70	2
	Yes, as nurse	77	2
	Yes, as doctor	24	1
	Yes, as other	292	7
	Prefer not to say	17	0
Higher risk for severe COVID-19 based on health-related risk factors	No	3,010	75
	Yes	981	24
	Prefer not to say	42	1
Perceived health status	Very good	782	19
	Good	2,334	58
	All right	736	18
	Bad	141	4
	Very bad	31	1
	Prefer not to say	9	0
Perceived allergy for vaccines	No, certainly not	1,310	33
	No, probably not	2,059	51
	Don’t know	572	14
	Yes, probably	63	2
	Yes, certainly	24	1
	Prefer not to say	5	0
Perceived previous infection with COVID-19	No, probably not	3,056	76
	Yes, probably	232	6
	Yes, certainly (tested positive)	225	6
	Don’t know	513	13
	Prefer not to say	7	0
Perceived role of religion or belief on vaccination decision	1 certainly not	2,897	72
	2	259	6
	3	206	5
	4	128	3
	5 certainly yes	138	3
	Unknown (all vaccinated respondents)	405	10
Total		4,033	100

Appendix Table 2. Partial dependence figures of the 10 most essential determinants by random forest analyses

Determinant	Values determinant	Corresponding values vaccination intention (partial dependence)
Vaccination, end of crisis	1	3.90
	2	3.97
	3	4.12
	4	4.27
	5	4.33
Vaccination, expectations of loved ones	1	4.01
	2	4.13
	3	4.30
	4	4.34
	5	4.42
Vaccination, developed too quickly	1	4.47
	2	4.47
	3	4.43
	4	4.34
	5	4.20
Vaccination, side effects well researched	1	4.07
	2	4.14
	3	4.30
	4	4.34
	5	4.34
Vaccination, approved therefore safe	1	3.92
	2	3.94
	3	4.08
	4	4.17
	5	4.22
Vaccination, good protection	1	4.18
	2	4.23
	3	4.35
	4	4.45
	5	4.45
Vaccination, new techniques are safe	1	4.18
	2	4.21
	3	4.32
	4	4.38
	5	4.40
Vaccination, live sooner without measures	1	4.15
	2	4.20
	3	4.25
	4	4.29
	5	4.31
Vaccination, behavior of loved ones	1	4.05
	2	4.14
	3	4.21
	4	4.26
	5	4.29
Vaccination, possibility of severe illness	1	4.39
	2	4.36
	3	4.33
	4	4.28
	5	4.26

	1	2	3	4	5	6	7	8	9	10	11	12	13	14	15	16	17	18	19	20	21	22	23	24	25
1	1.00	0.23	0.48	0.30	0.31	0.07	0.09	0.04	-0.04	0.07	-0.03	0.06	0.07	0.01	0.05	0.071	0.07	0.10	0.02	-0.15	-0.12	0.08	0.12	0.05	-0.00
2		1.00	0.16	0.29	0.17	0.02	0.03	0.16	-0.15	0.16	-0.12	0.17	0.16	-0.01	-0.03	0.08	-0.04	0.24	-0.32	-0.53	-0.23	0.19	0.24	0.13	-0.02
3			1.00	0.44	0.33	0.08	-0.05	0.07	-0.07	0.12	-0.06	0.09	0.14	0.02	0.04	0.13	0.15	0.16	-0.04	-0.13	-0.15	0.15	0.16	0.12	-0.05
4				1.00	0.36	0.09	-0.12	0.18	-0.18	0.23	-0.15	0.20	0.23	-0.03	0.02	0.19	0.18	0.28	-0.18	-0.23	-0.23	0.26	0.27	0.18	-0.11
5					1.00	0.06	-0.09	0.15	-0.13	0.17	-0.08	0.15	0.18	-0.03	-0.04	0.17	0.14	0.20	-0.07	-0.13	-0.17	0.17	0.18	0.14	-0.06
6						1.00	0.26	-0.18	0.19	-0.14	0.12	-0.17	-0.15	0.17	0.15	-0.08	-0.02	-0.11	0.02	0.00	0.05	-0.06	-0.08	-0.02	0.08
7							1.00	-0.57	0.55	-0.57	0.49	-0.59	-0.56	0.30	0.24	-0.40	-0.30	-0.50	0.21	0.09	0.39	-0.42	-0.42	-0.23	0.35
8								1.00	-0.68	0.73	-0.49	0.76	0.64	-0.31	-0.28	0.44	0.27	0.58	-0.23	-0.18	-0.40	0.47	0.48	0.26	-0.32
9									1.00	-0.69	0.52	-0.69	-0.60	0.36	0.27	-0.44	-0.29	-0.54	0.24	0.20	0.42	-0.46	-0.47	-0.26	0.30
10										1.00	-0.51	0.75	0.68	-0.30	-0.27	0.48	0.32	0.61	-0.25	-0.19	-0.45	0.50	0.52	0.30	-0.33
11											1.00	-0.53	-0.48	0.25	0.24	-0.35	-0.24	-0.44	0.22	0.17	0.41	-0.40	-0.40	-0.24	0.25
12												1.00	0.74	-0.31	-0.29	0.50	0.32	0.64	-0.25	-0.18	-0.44	0.53	0.54	0.31	-0.33
13													1.00	-0.33	-0.29	0.53	0.38	0.66	-0.26	-0.17	-0.44	0.56	0.55	0.36	-0.33
14														1.00	0.25	-0.20	-0.14	-0.27	0.09	0.08	0.20	-0.19	-0.20	-0.10	0.22
15															1.00	-0.26	-0.16	-0.28	0.07	0.06	0.19	-0.17	-0.19	-0.11	0.19
16																1.00	0.58	0.57	-0.18	-0.14	-0.35	0.44	0.46	0.29	-0.24
17																	1.00	0.40	-0.08	-0.02	-0.19	0.35	0.32	0.27	-0.20
18																		1.00	-0.28	-0.26	-0.47	0.61	0.60	0.36	-0.30
19																			1.00	0.37	0.33	-0.24	-0.25	-0.14	0.16
20																				1.00	0.30	-0.21	-0.24	-0.10	0.09
21																					1.00	-0.41	-0.42	-0.23	0.25
22																						1.00	0.72	0.47	-0.26
23																							1.00	0.39	-0.24
24																								1.00	-0.15
25																									1.00

Appendix Figure 1. Coronavirus disease (COVID-19) vaccination intent and belief that vaccination will end the pandemic. Detailed correlation matrix (corresponding to Figure 1 in main text). Pearson's correlation matrix (2-tailed) heat map with all beliefs about COVID-19 and COVID-19 vaccinations visualized per mental models element (risk perceptions COVID-19: self, risk perceptions COVID-19: loved ones, safety vaccination, effectiveness vaccination, (social) benefits vaccination, alternatives to vaccination, social norms vaccination behavior, accessibility vaccination).

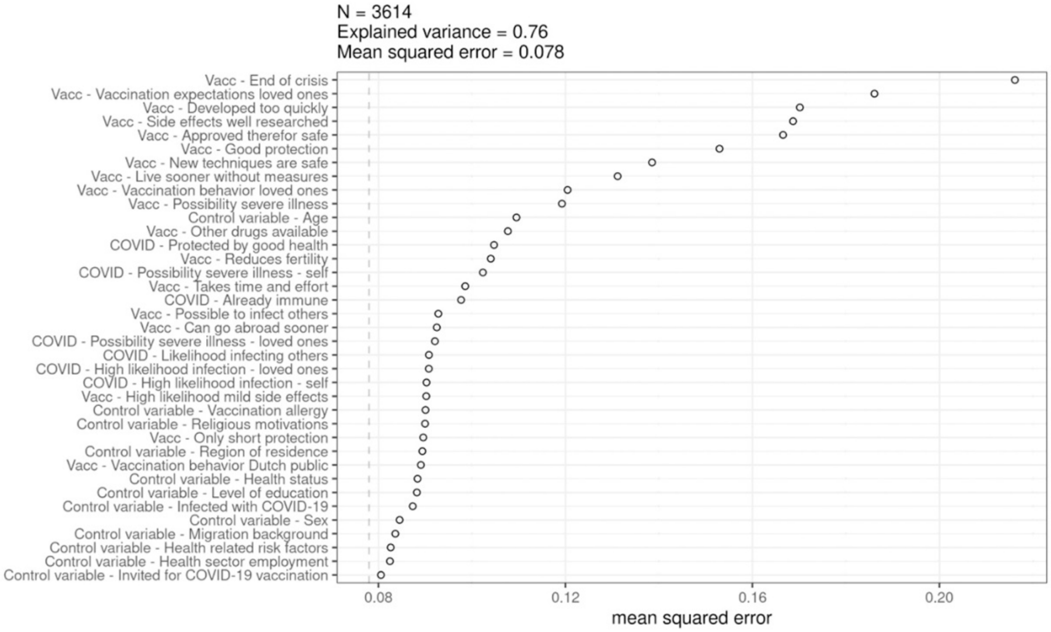

Appendix Figure 2. Variable ranking in random forest model with all determinants. Dashed vertical line indicates mean squared error (0.078). COVID-19, coronavirus disease; Vacc, vaccination.
